# *ZmbZIP39*: orchestrating source, flow, and sink for maize grain development

**DOI:** 10.1093/plcell/koag070

**Published:** 2026-03-16

**Authors:** Hongwei Jing

**Affiliations:** Assistant Features Editor, The Plant Cell, American Society of Plant Biologists; Department of Horticultural Science, North Carolina State University, Raleigh, NC 27695, United States

Photosynthesis is a fundamental biological process through which plants convert sunlight, water, and carbon dioxide into oxygen and carbohydrates. These carbohydrates are either stored as starch or transported as sucrose, serving as the primary energy source for maintenance, metabolism, and growth throughout the plant life cycle (Braun 2022). Sucrose is the principal form of sugar translocated via the phloem, moving from photosynthetic source tissues, such as leaves, to heterotrophic sink tissues, including roots, stems, fruits, and seeds (Aluko et al. 2021). Efficient sucrose transport is particularly critical for seed development, which relies heavily on the transfer of carbon resources from maternal to filial tissues. Maize produces larger grains than most other cereal crops, reflecting its high efficiency in sucrose loading and transport to developing kernels (Bihmidine et al. 2013). However, the mechanisms by which photosynthesis at source tissues, phloem-mediated sucrose transport, and sucrose metabolism and nutrient storage at sink tissues are coordinated to satisfy the substantial sugar demand during maize grain filling remain poorly understood.

*ZmSUT1* (*Zea mays Sucrose Transporter 1*) is a crucial sucrose transporter that functions as a reversible carrier, mediating both sucrose uptake (loading) in source leaves and sucrose release (unloading) into sink tissues (Baker et al. 2016). In recent work, **Tao Yang and colleagues** (Yang et al. 2026) used the sucrose transporter *ZmSUT1* as a molecular target and discovered that *bZIP* transcription factor *ZmbZIP39* acts as a central coordinator, simultaneously regulating photosynthesis (source), sucrose transport during translocation (flow), and sucrose metabolism and nutrient storage (sink) (Fig. 1). Transactivation activity analyses and DNA-protein pulldown assays suggested that *ZmbZIP39* specifically binds to the G2 motif in the promoters of *ZmSUT1* and *ZmSUT7*. Further evidence for a direct interaction was provided by electrophoretic mobility shift assays and dual-luciferase reporter assays, confirming the critical role of *ZmbZIP39* in activating the expression of *ZmSUT1* and *ZmSUT7*. More experiments suggested that *ZmbZIP39* undergoes auto-transactivation by directly binding the G-box element in its own promoter. Collectively, these findings indicate that *ZmbZIP39* directly regulates *ZmSUT1* and *ZmSUT7*-mediated sucrose transport to facilitate phloem loading and unloading.

**Figure 1:**
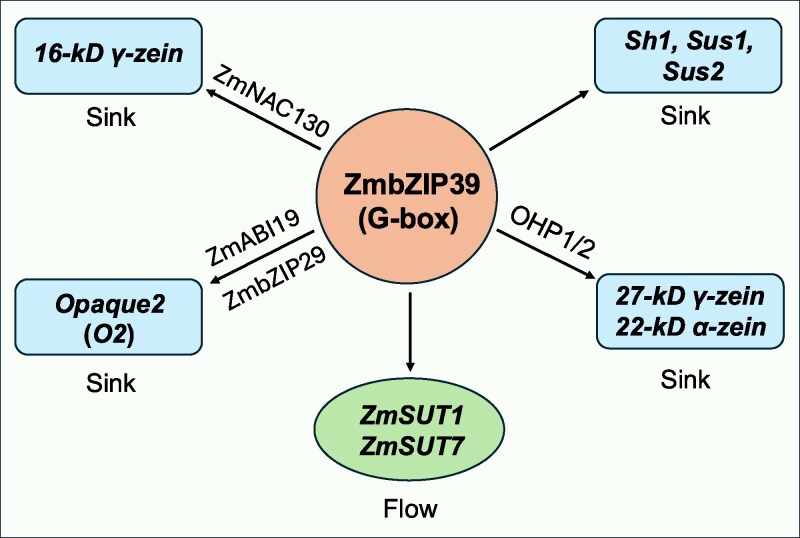
*ZmbZIP39* acts as a central regulator of source-flow-sink relations during maize grain filling. *ZmbZIP39* directly regulates expression of the sucrose transporters *ZmSUT1* and *ZmSUT7*, enhancing phloem loading and unloading through the flow-based pathway. *ZmbZIP39* also directly regulates 3 sucrose synthase (*SUS*) genes, *Sh1*, *Sus1*, and *Sus2*, promoting sucrose metabolism in sink tissues. Additionally, *ZmbZIP39* cooperates with *ONE-HELIX PROTEIN1* (*OHP1*) and *ZmNAC130*, synergistically enhancing the expression of 3 *zein* genes, *22-kD α-zein*, *27-kD γ-zein*, and *19-kD α-zein*, thereby strengthening sink storage capacity. Moreover, *ZmbZIP39* functions synergistically with *ZmABI19* and *ZmbZIP29*, promoting sink accumulation by activating expression of the endosperm-filling central gene *Opaque2* (*O2*). Figure credit: H. Jing.

*ZmbZIP39* gene knockout lines exhibited significant phenotypic defects, including reduced plant growth and decreased kernel length and mass. Conversely, overexpression of *ZmbZIP39* promoted taller plants with larger ears and increased ear mass. RNA-seq analyses showed that loss of *ZmbZIP39* disrupts transcriptome dynamics in source leaves and sink kernels. Combined transcriptome analysis, dual-luciferase reporter assays, and enzyme activity measurements suggested that *ZmbZIP39* directly activates the expression of 3 sucrose synthase (*SUS*) genes, *Sh1*, *Sus1*, and *Sus2*, thereby enhancing *SUS* activity and promoting sucrose metabolism in sink tissues. Interestingly, further evidence indicates that *ZmbZIP39* cooperates with *ONE-HELIX PROTEIN1* (*OHP1*) and *ZmNAC130*, synergistically enhancing the expression of *zein* genes, *22-kD α-zein*, *27-kD γ-zein*, and *19-kD α-zein*, thereby strengthening sink storage capacity. Additionally, *ZmbZIP39* functions synergistically with 2 endosperm-filling initiators, *ZmABI19* and *ZmbZIP29*, to promote sink accumulation by activating the expression of the endosperm-filling central gene *Opaque2* (O2).

Yang et al. (2026) identified *ZmbZIP39* as an important regulator of source-flow-sink relationships in maize (Fig. 1). However, several key mechanistic questions remain unresolved. For instance, protein sequence alignment revealed that ZmbZIP48 shares over 88% sequence identify with ZmbZIP39, yet *ZmbZIP48* transcript is expressed at extremely low levels across tissues and only weakly affects the transactivation of *ZmSUT1* and *ZmSUT7*. The reasons why this highly conserved ZmbZIP48 exhibits reduced regulatory activity warrant further investigation. In addition, the molecular mechanisms by which *ZmbZIP39* regulates sucrose synthase gene expression and interacts with *ZmABI19* and *ZmbZIP29* to synergistically activate *O2* remain to be elucidated. Addressing these questions will provide crucial insights for breeding high-yield and high-quality maize in the future.

## Recent related articles in *The Plant Cell*:

- Chen and coauthors (Chen et al. 2023) identified that transcription factors *ZmNAC128* and *ZmNAC130*, acting in cooperation with *O2*, directly regulate many genes associated with nutrient uptake and zeins and starch synthesis during endosperm filling in maize.
- Liu and coauthors (Liu et al. 2025) uncovered a key role for raffinose metabolism in controlling sugar allocation during embryo development and seed filling, highlighting the importance of carbohydrate partitioning for sink strength in cucumber.
- Wu and colleagues (Wu et al. 2023) demonstrated 3 key transcription factors, NAKED ENDOSPERM1 (NKD1), NKD2, and O2, interact to coordinately regulate gene networks controlling the transition from cellular development to grain filling in the maize endosperm, thereby restricting cell proliferation and differentiation while promoting nutrient biosynthesis.
- Zhang and colleagues (Zhang et al. 2025) identified regulatory mechanisms governing cotton fiber development, illustrating how transcriptional control of sink tissues can be leveraged for crop improvement.

## Data Availability

No new data were generated or analysed in support of this research.

## Dive Curated Terms

The following phenotypic, genotypic, and functional terms are of significance to the work described in this paper:

Opaque2 Gramene: Zm00001d018971
Opaque2 Araport: Zm00001d018971

Sus2 Gramene: GRMZM2G318780
Sus2 Araport: GRMZM2G318780

ZmNAC130 Gramene: Zm00001eb334180
ZmNAC130 Araport: Zm00001eb334180

Sus1 Gramene: GRMZM2G152908
Sus1 Araport: GRMZM2G152908
